# Ecotoxicological Differences of Antimony (III) and Antimony (V) on Earthworms *Eisenia fetida* (Savingy)

**DOI:** 10.3390/toxics11030230

**Published:** 2023-02-27

**Authors:** Jing Bai, Dan Lu, Linyu Chen, Weiying Liu, Yu Zheng, Guohong Xiang, Guiyuan Meng, Zhong Lin, Renyan Duan

**Affiliations:** 1 College of Agriculture and Biotechnology, Hunan University of Humanities, Science and Technology, Loudi 417000, China; 2 Hunan Key Laboratory of Ecological Remediation of Antimony Mine, Loudi 417000, China; 3 College of Chemistry and Environmental Sciences, Guangdong Ocean University, Zhanjiang 524088, China

**Keywords:** antimony, earthworm, acute toxicity, aging processes, speciation

## Abstract

In this study, we assessed the acute and chronic toxic effects of Sb (III) and Sb (V) on *Eisenia fetida* (Savingy) (*E. fetida*) by applying the filter paper contact method, aged soil treatment, and avoidance test experiment. In the acute filter paper contact test, the LC50 values for Sb (III) were 2581 mg/L (24 h), 1427 mg/L (48 h), and 666 mg/L (72 h), which were lower than Sb (V). In the chronic aged soil exposure experiment, when the Sb (III)-contaminated soil was aged 10 d, 30 d, and 60 d after exposure for 7 d, the LC50 value of *E. fetida* was 370, 613, and >4800 mg/kg, respectively. Compared to Sb (V) spiked soils aged only for 10 d, the concentrations causing 50% mortality significantly increased by 7.17-fold after 14 days of exposure in soil aged for 60 d. The results show that Sb (III) and Sb (V) could cause death and directly affect the avoidance behavior of *E. fetida*; yet, the toxicity of Sb (III) was higher than that of Sb (V). Consistent with the decrease in water-soluble Sb, the toxicity of Sb to *E. fetida* was greatly reduced with time. Therefore, in order to avoid overestimating the ecological risk of Sb with varying oxidative states, it is important to consider the forms and bioavailability of Sb. This study accumulated and supplemented the toxicity data, and provided a more comprehensive basis for the ecological risk assessment of Sb.

## 1. Introduction

Antimony (Sb) is a toxic element that survives once released into local ecosystems [1]. Sb can enter the human body by different routes, including skin contact, respiration, ingestion, etc. Once inside, it affects protein metabolism and damages the liver, heart, respiratory, and nervous system [2,3]. Local environments around Sb mining and smelting areas in China are seriously contaminated [4]. The maximum acceptable value of Sb in soil regulated by the World Health Organization (WHO) is 36 mg/kg [5]. However, our previous studies showed that due to untreated discharge and waste residues, the soil Sb concentration in the “World Antimony Capital” Xikuangshan Mine could reach 8591.16 mg/kg [6].

Sb is primarily presented as trivalent (Sb (III)) and pentavalent (Sb (V)) forms in environmental, biological, and geochemical samples [7]. Inorganic Sb was found to be more toxic than organic ones, while Sb (III) is more toxic than Sb (V) [8]. Sb (V) is the predominant form that exist as Sb (OH)_6_^-^ in oxic environments, while Sb (III) primarily exhibits as Sb(OH)_3_ and is more stable and toxic under anaerobic conditions [7,9]. Sb (III) has a more crucial role than Sb (V) in the movement and toxicity assessment as it is more easily adsorbed on the surface of metals and minerals; it can also oxidize to Sb (V) in the soil environment to change the total toxicity level [10,11,12]. Environmental management and risk assessment of soil pollution are both based on standards for soil environmental quality. The present soil environmental quality guidelines for agricultural land in China are set as a result of the lack of toxicity thresholds based on ecological consequences. Therefore, both the total concentration and the Sb forms need to be analyzed when evaluating the toxicity of Sb.

Most experiments adopted a short-term exogenous addition of Sb to the soil to simulate the Sb pollution in the field; yet, these experiments did not consider the aging effect of Sb in the soil. After entering the soil, water-soluble heavy metals rapidly complete the solid–liquid distribution and a series of reactions, such as adsorption and desorption, redox, dissolution and precipitation, and complexation and chelation within soil colloids; moreover, they also begin a slow aging process [13]. Therefore, metal aging in soil is treated as an important factor influencing its availability and toxicity to organisms [14,15]. In general, water-soluble speciation of heavy metal is easily transformed into residual speciation [16]. Studies also confirmed that the aging process has a key role in the ecological toxicity of heavy metals, and there is a significant difference in the effectiveness and toxicity compared with artificially added heavy metals in polluted soil [17,18,19]. Moreover, unlike other heavy metals, such as copper, zinc, cadmium, and lead, which do not undergo redox reactions in soil, the mobility and toxicity of Sb depend on its speciation, i.e., its oxidation states [20]. As a result, using field soil test results to represent the actual situation of Sb pollution in natural soil is not accurate enough. Therefore, when evaluating soil Sb (III) and Sb (V) ecotoxicity and providing basic toxicological data for the formulating of soil Sb quality standards, it is particularly important to consider the aging process to avoid overestimating its ecological risk.

As increasing attention is being paid to the toxicity of Sb and its environmental risks, some studies were reported on the toxicity of Sb to plants [8,21] and microorganisms [22,23], but little information is available on the Sb risk assessment to terrestrial invertebrates. As a representative of soil invertebrates, earthworms have an important role in evaluating the pollution of ecosystems. Earthworms can enhance soil texture, stimulate organic matter decomposition, boost carbon and nitrogen cycles, and keep soil permeability and moisture levels [24]. In addition, earthworms generate a drilosphere system that affects soil pH, redox potential, soluble carbon, chelate concentration, and other parameters, consequently affecting the biological toxicity, movement, and adsorption of pollutants [25,26].

*Eisenia fetida* (Savingy) (*E. fetida*) is used as a model system to study the toxic effects of metals and organic pollutants [27,28]. However, so far, only one study quantified the soil Sb (III) toxicity to *E. fetida* before and after the aging process [29], while acute and chronic toxicity of different Sb speciation on *E. fetida*, especially the toxicological parameters, are still not fully understood.

In this study, we quantified the toxicity of different soil Sb forms to earthworm *E. fetida*. The objectives of this study included: (1) study of the toxic effect of Sb (III) and Sb (V) on *E. fetida* by monitoring the morphological abnormalities and avoidance response of *E. fetida*; (2) exploring the acute toxic effects of two forms of antimony to *E. fetida* by investigating the dynamic dose–response relationship between earthworm mortality and Sb (III) and Sb (V) treatment levels; and (3) assessing the chronic toxic effect of the aging process on the toxicity and speciation by analyzing the Sb speciation and its relationship with mortality. These data may provide a scientific basis for the ecological risk assessment of soil Sb and data support for formulating soil Sb environmental quality standards.

## 2. Materials and Methods

### 2.1. Experimental Soils, Earthworms, and Chemicals

The experimental soil was collected from the top layer (0–20 cm) at the Jiu Er Base (27°46′27″ N, 112°1′30″ E) of Hunan University of Humanities, Science and Technology in Loudi, Hunan, China. The soil had a mechanical composition of sand 38.72%, silt 33.36%, and clay 27.92%, and was classified according to the United States Department of Agriculture soil texture triangle standard. The collected fresh soil was sieved with a 2 mm mesh and stored in the dark at 4 °C for 1 week until use. Table 1 lists the tested field soil’s main physical and chemical characteristics. Soil pH was measured using a soil/water ratio of 1:2.5. Organic matter content was measured by the dichromate oxidation procedure. Total nitrogen and available nitrogen were determined by an elemental analyzer (Vario EL III, Elementar). Total phosphorus and available phosphorus were measured by ammonium molybdate by a UV–visible spectrophotometer (SP2500, Spectrum). Total potassium and available potassium were determined by flame photometry after extraction with sodium hydroxide and ammonium acetate, respectively [30]. Soil mechanical composition was determined by the pipette method based on the American classification standards [31].

The experimental earthworm *Eisenia fetida* (Savingy) (*E. fetida*) was chosen as the model animal [32] and purchased from Wangjun Earthworm Base, Jurong, Jiangsu Province. Healthy mature adult earthworms (with a mass of 400 to 600 mg) with well-developed clitella were randomly selected before experimentation. Sb (III) and Sb (V) test reagents were antimony potassium tartrate (C_4_H_4_KO_7_Sb·½H_2_O, CAS Number: 28300-74-5) and potassium hexahydroxoantimonate (KSb(OH)_6_, CAS Number: 12208-13-8), respectively. All chemicals were of analytical grade and used without further purification.

### 2.2. Toxicity Assessments Assay

#### 2.2.1. Acute Contact Test

A filter paper contact test was performed to preliminarily estimate the percutaneous toxicity of antimony [33]. *E. fetida* were placed on wet filter paper to eliminate gut contents in an incubator for 24 h before the test (20 ± 1 °C, relative humidity of 70%). The filter paper was then soaked in 1 mL of a solution containing Sb (III) or Sb (V) in concentrations ranging among 0, 80, 160, 320, 640, 960, 1280, 1600, 1920, 2560, 3200, 4160, and 5120 mg/L; ultrapure water was used for the control experiment. Each Petri dish contained 1 earthworm, and each concentration had 10 replicates. All Petri dishes were sealed with microperforated plastic wrap and cultivated at 20 ± 1 °C in the dark. After 24, 48, and 72 h of exposure, the mortality of *E. fetida* was analyzed based on the response to the mechanical stimulus using a needle. Furthermore, *E. fetida* was photographed in their typical morphological variations. LC50 was calculated after 24, 48, and 72 h exposure.

#### 2.2.2. Earthworm Avoidance Test

Avoidance refers to earthworm migration from contaminated soil to clean soil. Briefly, 1 kg soil was placed in polyvinyl chloride soil columns (height 15.3 cm × upper diameter 19 cm × bottom diameter 13.3 cm), which were divided equally into two sections: one section was filled with 500 g of control soil, while the other half used 500 g soil which was contaminated with different Sb (III) and Sb (V) concentrations (0, 50, 100, 200, 400, and 800 mg/kg). After soil preparation, twenty *E. fetida* were placed at the intersection, and the soil columns were sealed with microperforated plastic wrap to avoid earthworm escape. Each concentration had three replicates and was completely randomized. After 48 h of exposure, the *E. fetida* in each soil section was counted. The value of net avoidance response (NAR) was used to quantify the avoidance behavior of earthworms using Equation (1):

(1) $$\mathrm{NAR}=\frac{\left(C-T\right)}{N}\times 100\%,$$

where C and T are the number of *E. fetida* in the control soil and spiked soil, respectively, while N is the total number of *E. fetida* per container, 20. NAR > 0 indicated avoidance, and NAR ≤ 0 indicated no avoidance or attraction to the spiked soil. NAR > 80% means a possible loss in habitat function [34].

#### 2.2.3. Chronic Toxicity Test

The soil test was performed to assess chronic toxicity. Soils with three aged lengths (10 d, 30 d, and 60 d) were used to evaluate Sb (III) and Sb (V) toxicity after the aging process. Each Sb (III) and Sb (V) concentration solution was mixed with 1 kg soil in polyvinyl chloride soil columns (height 12.5 cm × upper diameter 14 cm × bottom diameter 10 cm) to obtain multiple soil samples, including Sb (III) in concentrations of 0, 400, 800, 1600, 3200, and 4800 mg/kg, and Sb (V) in concentrations of 0, 2400, 4800, 9600, 14,400, and 19,200 mg/kg. All treatments were conducted in four replicates. The soil columns were equilibrated for 10 d, 30 d, and 60 d in a temperature incubator (20 ± 1 °C), then investigated with *E. fetida*.

*E. fetida* was pre-treated by washing with deionized water and placing on wet filter paper at 20 ± 1 °C in the dark for 24 h to defecate, then incubated in the uncontaminated soil for one month before use. Once the aged soil was ready, *E. fetida* was filled at the density of 20 individuals/kg soil. The soil columns were covered with microperforated plastic wrap to avoid *E. fetida* escape. Tests were carried out under controlled temperature (20 ± 1 °C), photoperiod (12 h:12 h), and soil water content (25%). All assays were also carried out for data comparison using control samples moistened with ultrapure water. The mortality of *E. fetida* was counted on day 2, 7, and 14 of exposure. LC50 was calculated after the 7th and 14th day.

### 2.3. Determination of Water-Soluble Sb Concentration

From each replicate of 10-day and 60-day-aged treatment, soil samples were air-dried before sieving (100-mesh) to remove roots and debris. Then, 2 g of each soil sample was added into a 50 mL plastic centrifuge tube, soaked with 20 mL of deionized water, mixed at 25 °C and 60 r/min for 2 h, and centrifuged at 4000 r/min. The supernatant was filtered through a 0.45 μm cellulose acetate filter [35]. A dual-channel atomic fluorescence photometer was used to calculate the total Sb (Beijing Haiguang instrument Co., AFS-2100, Beijing, China). Sb had a detection limit of 0.010 mg/kg.

### 2.4. Statistical Analysis

A date was expressed as the means ± standard deviation (SD) and processed in Microsoft Excel. One-way ANOVA was performed at the *p* < 0.05 confidence level using Duncan’s multiple range test with SPSS 16.0 software. The LC50 for *E. fetida* was determined using the earthworm mortality data and the dose–response (GraphPad Prism 8.0). All figures of results were performed using Origin 2019.

## 3. Results

### 3.1. Behavior and Morphological Changes in Earthworms during the Toxicity Tests

In the filter paper contact test, morphological abnormalities were observed in the *E. fetida* exposed to Sb (III) (Figure S1a–d) and Sb (V) (Figure S1e–h). No obvious abnormal behavior of earthworms was detected at the concentration of 320 mg/L of Sb (III) and Sb (V), while severe physical stress occurred with Sb (III) ≥ 1280 mg/L and Sb (V) ≥ 2560 mg/L at 24 h (Figure S1a,e); the symptoms included yellow coelomic fluid secretion, and partial diminution in Sb (III) (3200 mg/L, 24 h) (Figure S1b). In addition, *E. fetida* lost the ability to wiggle and fold on the surface of the tail when incubated with Sb (V) (5120 mg/L, 24 h) (Figure S1f). In Sb (III) (1280 mg/L, 48 h, Figure S1c) and Sb (V) (1280 mg/L, 48 h, Figure S1g), the *E. fetida* showed decoloration and broken multiple segmentation symptoms, and the discontinuous and granular tail ends could be easily torn apart; general symptoms included segmentation, cracks, and atrophy on clitellum (Figure S1b). It was worth noting that the earthworm survived 72 h in Sb (V) (1600 mg/L), merely showing abnormal swelling (Figure S1h). Sb (III) and Sb (V) seem to influence the morphological feature of earthworms. Mucous secretion and segmentation were common in most of the exposed *E. fetida* in the present study. The overall damage degree of earthworms was strongly connected with increasing exposure level and duration, i.e., the greater the antimony stress intensity, the longer the contact time, and the more serious the morphological abnormalities.

### 3.2. Mortality and Dose-Response Relationship in Acute Contact Toxic Test

Next, the dose–response relationship of Sb (III) and Sb (V) toxicity on *E. fetida* was assessed. The curves were plotted with Sb (III) and Sb (V) concentration as the abscissa and *E. fetida* mortality as the ordinate. The goodness of fit was described by the Adj. coefficient of determination (Adj. R-Square).

**Figure 1:**
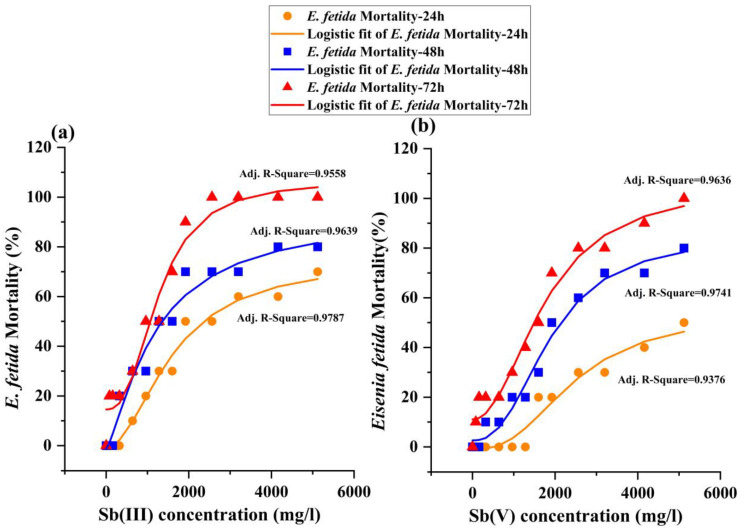
Mortality dose–response curves of *E. fetida* exposed to Sb (III) (**a**) and Sb (V) (**b**) for 24, 48, and 72 h by a filter paper contact toxic test. The Sb (III) and Sb (V) concentrations were prepared as 0, 80, 160, 320, 640, 960, 1280, 1600, 1920, 2560, 3200, 4160, and 5120 mg/L.

Figure 1 shows that Sb (III) and Sb (V) were toxic. The mortality of *E. fetida* increased with increasing heavy metal concentrations. The mortality rate in Sb (III) was generally higher than Sb (V) under the same condition. For example, at 24 h of exposure, the mortality rate was 50% in the Sb (III) 1920 mg/mL treatment group (Figure 1a), while it was only 20% in the Sb (V) 1920 mg/mL treatment group (Figure 1b); at 48 h of exposure, its was 70% in the Sb (III) 1920 mg/mL treatment group (Figure 1a) and 50% in the Sb (V) 1920 mg/mL treatment group (Figure 1b). All earthworms died after 72 h of exposure under Sb (III) 2560 mg/mL treatment and Sb (V) 5120 mg/mL treatment.

The LC50 values for *E. fetida* were determined using the data analysis regression probit procedure. The LC50 with a 95% confidence interval in filter paper contact treatments is provided in Table 2. For earthworms treated with Sb (III) in the filter paper contact test, the LC50 was 666 mg/L with 72 h exposure, which is approximately 0.74-fold lower than at 24 h (2581 mg/L). The longer the exposure duration, the smaller the LC50 was. The same trend was also observed in the Sb (V) treatment. LC50 values for *E. fetida* exposed to Sb (V) for 24, 48, and 72 h were 4675, 2223, and 1126 mg/L, respectively. The 24 h-LC50 in Sb (V) (4675 mg/L) was approximately 1.81-fold that in Sb (III) (2581 mg/L), while the 72 h-LC50 values were 1126 mg/L and 666 mg/L in the Sb (V) and Sb (III), respectively. These results indicate that Sb (III) is more toxic to *E. fetida* than Sb (V) in acute tests.

### 3.3. Avoidance Responses

**Figure 2:**
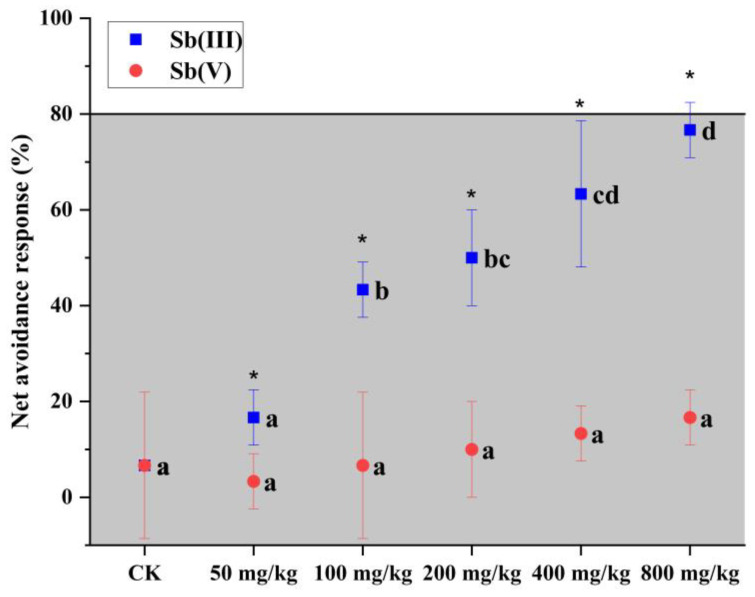
Net avoidance test using earthworm *E. fetida* exposed to Sb (III) and Sb (V) treatment. NAR > 80% indicate a possible loss in habitat function. Data were reported as mean ± standard deviation (M ± SD, *n* = 3). Significant differences in the same treatment were marked with different lowercase letters at *p* < 0.05. Significant differences between Sb (III) and Sb (V) of the same concentration level were marked with an asterisk at *p* < 0.05.

As shown in Figure 2, a standardized avoidance test was carried out to investigate the avoidance response of *E. fetida* exposed to soil treated with Sb (III) and Sb (V). Briefly, the values of NAR gradually increased with the increases in treatment levels (for both Sb (III) and Sb (V) treatment), indicating a good dose–response relationship. *E. fetida* exposed to Sb (V) displayed non-significant avoidance responses (*p* > 0.05), while the soil indicated a more obvious decline of habitat function with higher Sb (III) concentration. NAR values in Sb (V) treatment were overall lower than that in Sb (III) treatment, among which a significant difference was found beginning with 50 mg/kg (*p* < 0.05), demonstrating that Sb (V) could reduce the avoidance response of *E. fetida*. Relatively mild avoidance responses were also observed in soil treated with Sb(V) < 800 mg/kg.

In addition, NAR values reached 76.67 ± 5.77% at 800 mg/kg of Sb (III) treatment, indicating that soil biological adaptation function was destroyed to a certain extent [28]. Meanwhile, NAR values in remaining treatment levels were in the range of 16.67–63.33%, indicating that even a low Sb (III) level was potentially harmful to earthworms and the soil environment.

### 3.4. Mortality and Aging Relationship in Chronic Toxic Test

**Figure 3:**
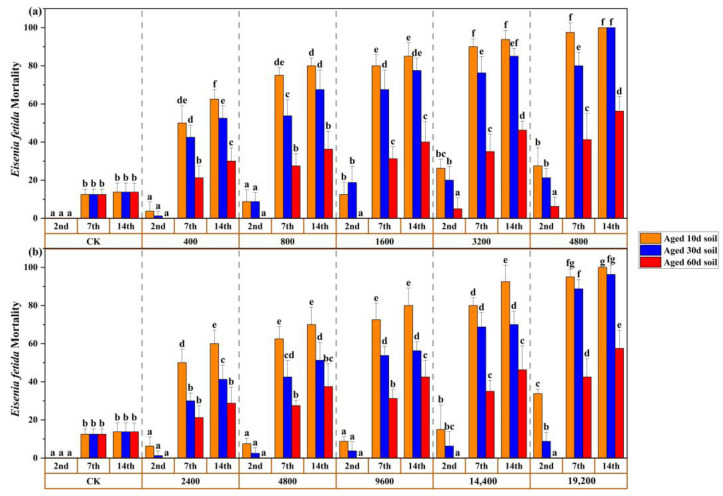
Mortality of *E. fetida* after 2 d, 7 d, and 14 d of exposure to Sb (III) (**a**) and Sb (V) (**b**) spiked soil aged for 10 d, 30 d, and 60 d. Error bars indicate standard deviations (n = 4). In the same dotted frame, bars with the different lowercase letter indicate significant differences between aging treatments at the same Sb (III) and Sb (V) concentration (*p* < 0.05). The Sb (III) concentrations were prepared as CK, 400, 800, 1600, 3200, and 4800 mg/kg. The Sb (V) concentrations were prepared as CK, 2400, 4800, 9600, 14,400, and 19,200 mg/kg.

A systematic investigation of the aging process to impact Sb toxicity on earthworms is shown in Figure 3. For 10-day-aged treatment of Sb (III) 1600, Sb (III) 3200, and Sb (III) 4800, within the three observation timeslots (2 d, 7 d, and 14 d), the mortality increased from 12.50% to 85.00%, from 26.25% to 93.75%, and from 27.50% to 100.00%, respectively (Figure 3a). No earthworms survived for more than 14 d of incubation in Sb (III) 4800. For 10-day-aged treatment of medium content (Sb (III) 400 and Sb (III) 800), the mortalities of earthworms were 3.75% and 8.75% at the first 24 h, respectively; however, persistent exposure was the determinant of the gradual death.

Compared with the 10-day-aged soil, the mortalities for the 60-day-aged Sb (III) soil samples were significantly reduced. With the Sb (III) 4800 mg/kg concentration, the mortality rate decreased from 27.50% to 6.25% (2 d), from 97.50% to 41.25% (7 d), and from 100% to 56.25% (14 d) (*p <* 0.05). In the remaining concentrations of 60-day-aged soil, the mortality rates of the earthworms were all < 50%. In addition, mortality significantly decreased (*p <* 0.05) in Sb (III) 1600 and Sb (III) 3200 at the end of 7 d and 14 d, indicating that Sb (III) toxicity was significantly diminished due to the aging process of soil.

Likewise, the effects of aging on *E. fetida* were measured in the Sb (V) toxicity test up to 2 d, 7 d, and 14 d of exposure (Figure 3b). After 7 days and 14 days of exposure in the 10-day-aged soil, the earthworm mortality (>62.5%) had no significant difference at Sb (V) 4800, Sb (V) 9600, and Sb (V) 19,200. For 60-day-aged treatment, earthworms did not die in the first 2 days at all concentrations; however, persistent exposure was the determinant of the gradual death. With the highest Sb (V) 19,200 treatment, the mortality at 2 d, 7 d, and 14 d significantly decreased from 33.75% to 0.00%, 95.00% to 42.50%, and 100% to 57.50% (*p <* 0.05), respectively.

LC50 values of Sb (III) and Sb (V) for *E. fetida* survival are shown in Table 3. LC50 values of Sb (III) treatment were decreased compared to that in Sb (V) treatment within aged 10 d, 30 d, and 60 d soil. For example, LC50 at 7 d and 14 d of exposure in aged 10 d soil increased from 370 to 2730 mg/kg, and from 254 to 2040 mg/kg, respectively. LC50 value of Sb (V) in aged 30 d soil was 6033 mg/kg and 4391 mg/kg at 7 d and 14 d of exposure, respectively, which was approximately 9.83 and 11.00-fold higher than in Sb (III), indicating that *E. fetida* was more sensitive to Sb (III) in the natural soil. LC50 values of Sb (III) and Sb (V) in aged 60 d soil were higher than the highest antimony concentration, which represented that Sb (III) and Sb (V) had no chronic toxicity to *E. fetida* within the concentration range.

### 3.5. Mortality and Water-Soluble Sb Relationship after the Aging Process

**Figure 4:**
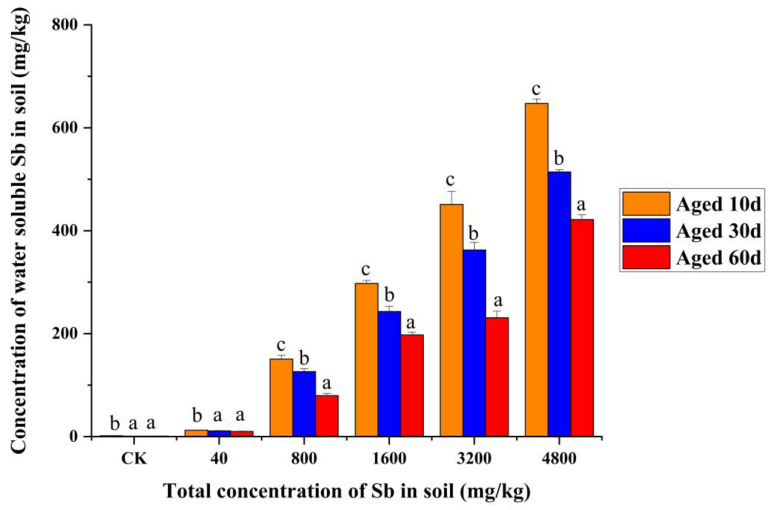
Water-soluble Sb concentrations in the Sb (III)-spiked soils aged for 10 d, 30 d, and 60 d. Error bars indicate the standard deviations (*n* = 4). Bars with the different lowercase letter indicate significant differences between aging treatments at the same Sb concentration (*p* < 0.05).

Concentrations of water-soluble Sb in aged 10 d, 30 d, and 60 d Sb (III) treatments are shown in Figure 4. After adding Sb (III) to the soil, with the prolongation of the aging time, the proportion of water-soluble Sb content in the total Sb in the soil with different concentrations of Sb significantly decreased. Briefly, the water-soluble Sb content reduced from 12.50 and 300.60 mg/kg to 647.36 and 421.73 mg/kg, respectively, at soil Sb (III) concentrations of 40 and 3200 mg/kg, and the reduction ratio was 79.88% and 51.17%, respectively. As shown in Figure S2, the mortality of earthworms was directly proportional to the decrease in soil water-soluble Sb concentration. The correlation coefficients were 0.969 and 0.935 in aged 10 d and 30 d soil, respectively (*p* < 0.01). These results indicate that the water-soluble Sb in soil decreases significantly during aging. Consistent with the changes in water-soluble Sb, the toxicity of Sb to *E. fetida* greatly reduced as the aging time was prolonged.

## 4. Discussion

### 4.1. Effects of Sb (III) and Sb (V) on the Morphological Variation in E. fetida

Sb (III) and Sb (V) negatively affected the morphological of *E. fetida* for 24 h to 72 h of exposure. Compared to lead, toxic symptoms of *E. fetida* caused by Sb were consistent with previous studies [36]. According to the morphology and behavior of Sb effects, we could confirm that filter paper contact treatment led to the highest toxicity and caused more pathological abnormalities. Toxic symptoms of Sb in the acute contact test were greater than in the chronic toxicity test, but both conditions led to a morphological change in earthworms.

### 4.2. Effects of Sb (III) and Sb (V) on the Mortality of E. fetida

This investigation used different exposure methods and periods to describe the negative impacts and differences between the two forms of Sb on *E. fetida*. The toxicity of Sb (III) was substantially higher than that of Sb (V) (Table 2 and Table 3). At the same exposure time, toxicity was inversely related to LC50 values, i.e., the lower the value, the greater the toxicity. The 24 h-LC50 in Sb (V) reached 4675.47 mg/L in the filter paper test, which was approximately 1.81-fold higher than that in Sb (III) (Table 2). Since there is limited study on Sb’s acute toxicity in *E. fetida*, it was discovered that Sb’s toxicity to other receptors is linked to its form variations. Some studies suggest aquatic organisms are more sensitive to Sb (III) than Sb (V) [37]. The toxicity of Sb (III) is 10 times that of Sb (V) in an aqueous solution [38]. Sb (III) and Sb (V) were discovered to have an impact on seed germination, root development, and yield [39]. If the yield is reduced by 10% as the “threshold level” of rice damage, the critical concentration of Sb (V) in the soil is about 300 mg/kg, while the critical concentration of Sb (III) is 150 mg/kg [40]. The toxicity differences between Sb forms are possibly related to variations in bioavailability and toxicity [41,42], but also differences in absorption, accumulation, transformation, and excretion mechanisms [43,44]. It was also reported that elevated Sb levels negatively affect the survival and casting activity of *Perionyx excavates* [45]. Only the toxic effects of different inorganic forms of Sb were evaluated in this study. In fact, Sb can be methylated under the action of the soil microbial community, and some studies suggest that Sb (III) is more inclined to methylation than Sb (V) [46]. This means that Sb is transformed from inorganic to organic, so it is necessary to further study the toxicity of Sb, especially antimony methylation, to earthworms.

### 4.3. Effects of Sb (III) and Sb (V) on the Avoidance Responses of E. fetida

According to the standard Organization for Standardization guidelines [34], only when the values of NAR exceed 80% of the habitat, soil function is destroyed by the contaminants. When *E. fetida* was exposed to 100 mg/kg of decabromodiphenyl ether and 500 mg/kg Pb, the value of NAR reached 53.33% [28]. After being exposed for 14 days to 1000 μg/kg of microcystins, *E. fetida* displayed no avoidance behavior [47]. In our study, the average NAR values in all Sb (III) treatments did not reach 80% but had a significant (*p* < 0.05) increase compared to the Sb (V) treatment, which indicates that Sb (III) may be potentially harmful to earthworms and their environment. However, Xu et al. reported that NAR values in Sb (III) 800 mg/kg, 1600 mg/kg, and 3200 mg/kg reached 93.33%, 100%, and 100%, respectively, for fresh treatment [29]. Compared to fresh treatment, the NAR of *E. fetida* caused by 10-day-aged soil Sb in our study was no more than the significant avoidance level, which indicated that the aging process had a great role in mitigating avoidance responses when the soil contained a high level of Sb (III).

### 4.4. Effects of the Aging Process on the Toxicity of Sb in Soil

Since filter paper tests can only evaluate the epidermis toxicity of antimony, further toxic mechanisms should be detected. Heavy metals can enter earthworms via skin penetration and ingestion [48]. Shao et al. used a filter paper contact test to evaluate the percutaneous toxicity of earthworms and a natural soil test to evaluate both percutaneous and oral toxicity [49]. Moreover, Zhong et al. confirmed that different agricultural soils affect the toxicity and bioavailability of Sb to *E. fetida* [50]. Therefore, soil chronic toxicity tests were performed in this study, and toxicity in different aging periods was compared. The results show that *E. fetida* mortality from Sb (III) and Sb (V) was dose and time-dependent, and heavily influenced by the substrate.

Mortality of earthworms in 60-day-aged treatment soil was reduced compared to that in 10 day treatment soil. For example, the mortality rate at 2 d in Sb (III) 4800 decreased from 27.50% to 6.25% (Figure 3). In the remaining concentration, earthworms survived for 2 d, and the mortality gradually increased at the end of 7 d and 14 d. In terms of measurement, some analysis consistent with our research revealed that Sb (III) toxicity was greatly reduced owing to soil-aged processes, and mortality induced by chronic stress was the most common cause of death [51]. In general, LC50 values increased with the increasing soil aging period (Table 3). Another study declared that when the copper in the soil was not aged, the EC50 for the reproduction of *Folsomia fimetaria* was 1300 mg/kg, and when the soil was aged after 21 weeks, EC50 reached 1850 mg/kg [52]. Many studies showed that aging mainly reduces the bioavailability or toxicity of heavy metals in soil by reducing their bioavailability [53]. Therefore, in the condition of Sb (III) and Sb (V) treatment, we could conclude that the LC50 values increase with aging, making antimony toxicity at lethal concentration decrease.

### 4.5. Effects of the Aging Process on Speciation of Sb in Soil

To understand the aging effect on the speciation of Sb in soil, we reported the changes in water-soluble speciation of Sb (III) and its chronic toxicity to *E. fetida* (Figure 4). It was concluded that the aging process of Sb leads to a decrease in earthworm mortality. At the same time, from the speciation, the essence of the aging process is a decrease in the proportion of water-soluble Sb. Chemical speciation affects metal solubility and toxicity [54]. Heavy metals are separated into five fractions, namely exchangeable, bound to carbonates, bound to iron and manganese oxides, bound to organic matter, and residual fractions [16]. The first fraction is the water-soluble and easily exchangeable metal considered the fraction potentially most available to living organisms. According to the extraction results, the concentration of water-soluble Sb decreased by 66.40%, 51.17%, and 65.17% of Sb (III)1600, Sb (III)3200, and Sb (III)4800 in the 60 d aging treatment compared with the 10 d aging treatment (Figure 4). This is consistent with prior research, which found that Sb has a strong attraction for crystalline and non-crystalline Fe oxides, as well as hydroxide minerals [55]. Mn minerals coupled with Fe oxides demonstrated considerable promise for immobilizing Sb via oxidation of Sb (III) and sorption of Sb (V) [20]. It is observed that the difference between before and after aging of the concentration of water-soluble Sb could reduce mortality, avoidance response, and pathological abnormalities of earthworms. Previous studies showed that soil properties, including clay, cation exchange capacity, organic matter, Fe oxides, and pH were important factors predicting heavy metal toxicity [56]. To further understand the relationships between aging effects and toxicity, more investigation comprising various types of soil is required.

For the purpose of assessing risks, prioritizing tasks, and establishing environmental quality standards for metal pollutants, toxicology data are crucial and of great importance. Our data indicate that aging could reduce the toxicity of heavy metals to *E. fetida*, because soil properties substantially influence the bioavailability of heavy metal expression [57]. It is worth noting that in the gut of earthworms, a large amount of organic matter is decomposed and converted into humic substances [58]. Humic acid, an important redox intermediate in the reduction reaction of microorganisms, may strongly adsorb Sb (III) [10,59]. It has various functional groups, such as the phenolic hydroxyl group, carboxyl group, and carbonyl group, thereby changing the activity of heavy metals [60]. Therefore, in future experiments, we should pay close attention to earthworm disturbance and physical, chemical, and microbiological changes in the soil as a result of digestion. The mechanism between these and Sb’s valence, bioavailability, and toxicity needs to be further explored.

## 5. Conclusions

The present study validated the toxicity of Sb (III) and Sb (V) to the earthworm *E. fetida*. These data are essential for understanding how Sb affects earthworm survival; hence, the mortality and LC50 values can accumulate and supplement toxicological data of soil Sb (III) and Sb (V), and they also provide a more comprehensive basis for ecological risk assessment of Sb. In acute and chronic toxicity testing, Sb (III) was found to be more hazardous to earthworms than Sb (V). Sb (V) has a certain toxic impact on the morphological abnormalities of earthworms in the given concentration range, whereas the toxicity of Sb (III) was more significant than that of Sb (V). As a result, when assessing the harmful impact of Sb, it is vital to consider both the total concentration and the form difference. In addition, the dose–response relationship revealed that the values of LC50 for earthworm mortality decline with aging, indicating that Sb (III) and Sb (V) have the most toxicity before soil aging. The speciation results indicate that the concentration of exchangeable fraction Sb could increase earthworm mortality. Therefore, in order to avoid overestimating the ecological risk of Sb with varying oxidation states, it is important to consider the effect of soil age. In addition, further research is needed to determine the mechanisms of the combined toxicological effects of Sb (III) and Sb (V) on earthworms.

## Figures and Tables

**Table 1:** Physicochemical characteristics of the natural field soil used in this study.

pH	Organic Matter (g/kg)	Total Nitrogen (g/kg)	Available Nitrogen (mg/kg)	Total Phosphorus (mg/kg)	Available Phosphorus (mg/kg)	Total Potassium (g/kg)	Available Potassium (mg/kg)	Soil Mechanical Composition			Total Sb (mg/kg)
								Sand/%	Silt/%	Clay/%	
7.28	30.34	1.77	128.53	1.06	23.63	15.79	185.15	20.12	35.80	44.08	3.57

**Table 2:** LC50 (mg/L, 95% confidence intervals) of Sb (III) and Sb (V) to *E. fetida* in acute contact toxic test.

Exposure Time	Sb (III)		Sb (V)	
	LC50	95% Confidence Interval	LC50	95% Confidence Interval
24 h	2581	2248–3022	4675	3824–6483
48 h	1427	1175–1723	2223	1920–2608
72 h	666	323–1071	1126	733–1627

**Table 3:** LC50 (mg/kg dry weight, 95% confidence intervals) of Sb (III) and Sb (V) to *E. fetida* in chronic soil toxic assay.

Tests	Exposure Time	Sb (III)		Sb (V)	
		LC50	95% Confidence Interval	LC50	95% Confidence Interval
Aged 10 d Soil	7 d	370	92–632	2730	582–4556
	14 d	254	31–482	2040	457–3416
Aged 30 d Soil	7 d	614	81–1142	6033	3540–8786
	14 d	399	99–680	4391	1746–6738
Aged 60 d Soil	7 d	>4800		>19,200	
	14 d	3547	2294–8222	14,640	9751–35,023

## Data Availability

We can provide raw data and code if needed.

## Supplementary Materials

The following supporting information can be downloaded at: https://www.mdpi.com/article/10.3390/toxics11030230/s1, Figure S1. Effect of Sb (III) and Sb (V) on the morphology of earthworm *E. fetida* in toxicity tests. Figure S2: The relationship between mortality of earthworm from different aging time and concentration of water-soluble Sb.
