# Supplementary material for: Ecotoxicological Differences of Antimony (III) and Antimony (V) on Earthworms Eisenia fetida (Savingy)
Source: Toxics. 2023 Feb 27;11(3):230. doi: 10.3390/toxics11030230 (PMC10056663; doi:10.3390/toxics11030230)
Supplement: Supplementary file 1 [file toxics-11-00230-s001.zip › Highlights.pdf]

Highlights

- The ecotoxicological effect of Sb (III) on *E. fetida* is more toxic than Sb (V).
- The toxicity of Sb (III) and Sb (V) was greatly reduced as soil aging time was prolonged.
- The exchangeable Sb could seriously impact the toxicity of Sb and explain the decrease of toxicity with time.
