# Supplementary material for: Ecotoxicological Differences of Antimony (III) and Antimony (V) on Earthworms Eisenia fetida (Savingy)
Source: Toxics. 2023 Feb 27;11(3):230. doi: 10.3390/toxics11030230 (PMC10056663; doi:10.3390/toxics11030230)
Supplement: Supplementary file 1 [file toxics-11-00230-s001.zip › Supplementary data.pdf]

Supporting information for Results

Figure S1. Effect of Sb (III) and Sb (V) on the morphology of earthworm *E. fetida* according to the toxicity tests.

Figure S2. The relationship between mortality of earthworm from different aging time and concentration of water-soluble Sb. (a) aged treatment for 10d, (b) aged treatment for 30d, (c) aged treatment for 60d.

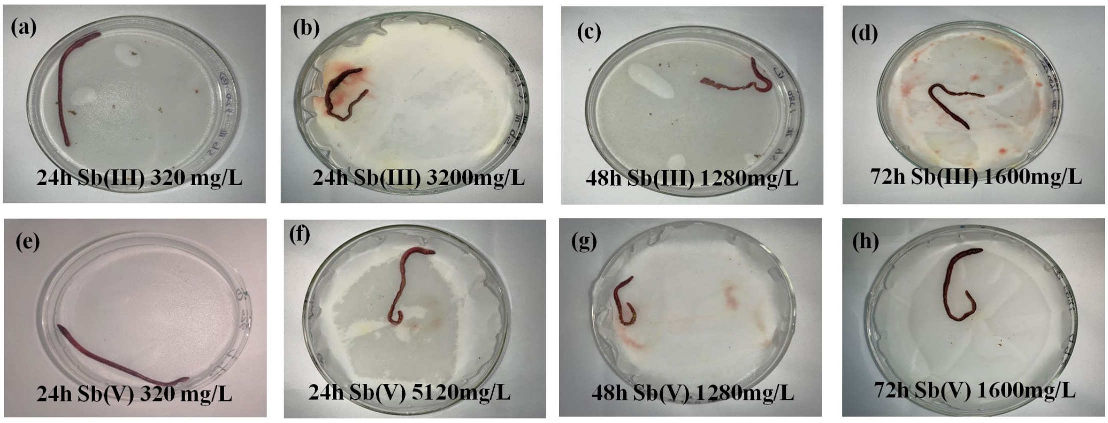

Figure S1. Effect of Sb (III) and Sb (V) on the morphology of earthworm *E. fetida* according to the toxicity tests.

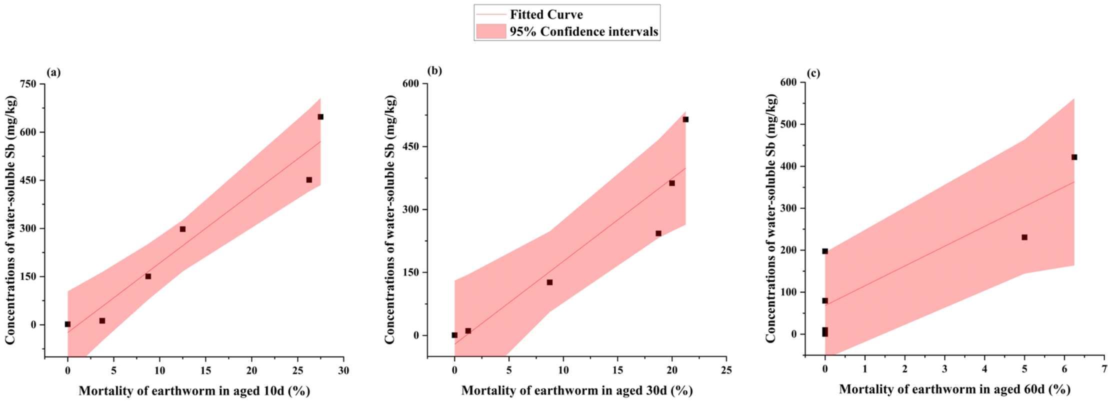

Figure S2. The relationship between mortality of earthworm from different aging time and concentration of water-soluble Sb. (a) aged treatment for 10d, (b) aged treatment for 30d, (c) aged treatment for 60d.
